# All these screens that we’ve done: how functional genetic screens have informed our understanding of ribosome biogenesis

**DOI:** 10.1042/BSR20230631

**Published:** 2023-07-07

**Authors:** Cecelia M. Harold

**Affiliations:** Department of Genetics, Yale School of Medicine, New Haven, CT, U.S.A.

**Keywords:** high-throughput screening, nucleolus, ribosome biogenesis, ribosomes, RNA

## Abstract

Ribosome biogenesis is the complex and essential process that ultimately leads to the synthesis of cellular proteins. Understanding each step of this essential process is imperative to increase our understanding of basic biology, but also more critically, to provide novel therapeutic avenues for genetic and developmental diseases such as ribosomopathies and cancers which can arise when this process is impaired. In recent years, significant advances in technology have made identifying and characterizing novel human regulators of ribosome biogenesis via high-content, high-throughput screens. Additionally, screening platforms have been used to discover novel therapeutics for cancer. These screens have uncovered a wealth of knowledge regarding novel proteins involved in human ribosome biogenesis, from the regulation of the transcription of the ribosomal RNA to global protein synthesis. Specifically, comparing the discovered proteins in these screens showed interesting connections between large ribosomal subunit (LSU) maturation factors and earlier steps in ribosome biogenesis, as well as overall nucleolar integrity. In this review, a discussion of the current standing of screens for human ribosome biogenesis factors through the lens of comparing the datasets and discussing the biological implications of the areas of overlap will be combined with a look toward other technologies and how they can be adapted to discover more factors involved in ribosome synthesis, and answer other outstanding questions in the field.

## Introduction

Ribosome biogenesis is an energetically costly and essential process responsible for manufacturing the protein-making machinery for the cell [1]. Briefly, this essential process begins with the transcription of the 47S polycistronic precursor ribosomal rRNA (pre-rRNA) in the nucleolus of eukaryotic cells. This rRNA will be processed and modified to eventually form the mature rRNA species known as the 5.8S, 28S, and 18S. A host of assembly factors and ribosomal proteins are involved in the ultimate assembly and joining of the mature 40S and 60S subunits of the ribosome. For decades, the detailed steps of ribosome biogenesis have been painstakingly elucidated by many groups in the yeast *Saccharomyces* *cerevisiae* due to the organism’s tractable genetics and wealth of tools already developed to map steps like pre-rRNA processing [2–6]. Even with such a solid groundwork laid out, the full elucidation of ribosome biogenesis in human cells has lagged behind its yeast counterpart. So how have we made progress in understanding human ribosome biogenesis?

The desire to identify proteins involved in ribosome biogenesis was driven in 1999 by the discovery of the association between mutated RPS19 and Diamond Blackfan anemia (DBA), which would become the first ribosomopathy [7]. These are a class of diseases caused by mutations in ribosome biogenesis factors. One such screening campaign as a later result of the discovery of DBA conducted by O’Donohue et al. specifically looked at the formation of the 40S upon siRNA knockdown of the 32 known small ribosomal proteins [8]. Interestingly, this study showed a difference in function between subsets of the ribosomal proteins in that one group strongly affected pre-rRNA processing and one group strongly affected maturation and export steps [8]. siRNA screens have already provided good information while leaving open several further avenues to explore, particularly to identify novel regulators of ribosome biogenesis.

Nearly ten years ago, the extent of high-throughput screening carried out to identify ribosome biogenesis factors consisted of an innovative screen performed in flies and yeast, which provided important insight on the conservation of factors involved in nucleolar form and function [9]. Neumuller et al. first used yeast to conduct a genome-wide analysis of factors affecting nucleolar size and fragmentation as indicators of RNA Polymerase I transcription [9]. This screen found 388 genes (∼6% of the yeast genome) with mutants that caused abnormal nucleolar size and/or fragmentation [9]. In order to assess the extent of evolutionary conservation, they performed a genome-wide RNAi screen in *Drosophila melanogaster* cells, using confocal microscopy to assess changes in nucleolar size [9]. This screen yielded 757 genes and confirmed that, like in yeast, the loss of the HIR (histone information regulator) complex resulted in increased rDNA transcription, as a highlighted example of conservation of complexes involved in ribosome biogenesis across species [9]. Overall, the basis for screening for ribosome biogenesis/nucleolar factors had a compelling base from which to start.

Around that time, genome-wide human cell screening was in beginning stages with a candidate screen from Kutay’s group [10]. Further screens were conducted to identify regulators of various stages of human ribosome biogenesis. These included a candidate screen looking for pre-rRNA processing factors [11], a genome-wide screen for 40S maturation factors [12], a genome-wide screen identifying proteins involved in changing nucleolar number [13] with a follow up in 2021 [14], and a genome-wide screen for 60S maturation factors [15]. Alongside these screens are two other important projects for establishing a full view of ribosome biogenesis, namely the human nucleolar proteome and the RNA polymerase I (RNAPI) interactome [16–18]. Even though we have made exceptional strides to push our understanding of human ribosome biogenesis forward, we still need to more fully understand this process.

Outside of and in conjunction with their major roles in protein production, the nucleolus and ribosome biogenesis are increasingly recognized as important modulators of human disease and as druggable targets. In 1896, when Pianese et al. first observed the connection between abnormal nucleoli and cancer, the importance of the nucleolus in cancer was put on the map [19]. Since then, the use of abnormal nucleoli as a prognostic indicator for poor outcomes in cancer has become standard [20,21]. Another danger of impaired ribosome biogenesis is the potential to develop a ribosomopathy. These diseases often display tissue-specificity and sometimes even predispose an individual to cancer later in life [22]. Examples include DBA and Treacher-Collins syndrome. The need to understand each regulator of ribosome biogenesis in humans is deeply apparent every time a patient is diagnosed with either a known or potentially new ribosomopathy [23].

This review describes our current understanding of ribosome biogenesis through the lens of functional genomics screening in human cells. Further, this article will discuss new technologies that are being applied or have the potential to be applied to extend our understanding of the underlying mechanism of human ribosome biogenesis in a robust, high-throughput manner. My aim is to clearly lay out the progress that has been made in understanding human ribosome biogenesis and highlight where more clarity could be useful. These discoveries and their molecular mechanisms might have profound implications for drug design and targeted therapies in the future for ribosomopathies, old and new, and cancer.

## The landscape of screens for proteins involved in human ribosome biogenesis

A significant challenge is to understand the conservation of ribosome biogenesis across life forms [24,25]. The simplest place to start looking for regulators in human cells is to examine proteins, which is why the aforementioned screens upon which this review will focus have centered on identifying protein regulators [10–15,17,18].

Screens can be classified as candidate or genome-wide, depending on the pool of targets that are screened. The main difference between candidate and genome-wide screens is that, like reverse genetic screens, candidate screens are screening against selected genes based on previously collected information, whereas a genome-wide screen typically uses a library of gene silencing tools (siRNA, CRISPR, etc.) to deplete known genes across the protein-coding genome. Of the screens discussed in this review article, Tafforeau et al. and Piñeiro et al., are candidate screens, while Badertscher et al., Dörner et al., and Farley-Barnes and McCann et al./Ogawa et al. are all genome-wide. Tafforeau et al. posed the question of how conserved is pre-rRNA processing between yeast and humans by conducting a candidate siRNA screen of nucleolar proteins [11]. This screen not only yielded 286 hits with processing phenotypes, but also showed that while 73% of the genes with known yeast homologs exhibited similar functions, 27% had functions unique or in addition to those known functions [11]. This demonstrated that although human pre-rRNA processing had great overlap with yeast, broader screening campaigns were necessary to fully understand human ribosome biogenesis. Piñeiro et al. similarly approached understanding the RNAPI interactome using a pseudo-candidate screen in that they looked specifically at the nuclear RNA interactome [18]. They used RNA interactome capture (RIC), which combines UV cross-linking, nuclei isolation, oligo(dT) capture, and mass spectrometry, in addition to RNA immunoprecipitations to identify 211 RNAPI interactors (datasets reviewed in Table 1) [18]. These 211 RNAPI interactors were specifically termed RNAPI-dependent RNA binding proteins (RBPs), as treatment with Actinomycin D (ActD), a known rRNA transcription inhibitor, reduced the binding of these proteins to the pre-rRNA. In contrast to these factors, 246 proteins did not show a reduction in binding following ActD treatment and were termed RNAPI-independent RBPs [18]. Candidates from the RNAPI-dependent RBPs category were further examined for subcellular localization and pre-rRNA binding in low-throughput assays [18]. Both candidate screens yielded important data on pre-rRNA transcription and processing in two different cell lines (cervical cancer HeLa cells for Tafforeau and breast epithelial MCF10A cells for Piñeiro).

**Table 1:** Screens identifying novel human ribosome biogenesis factors or interactors

Screen	Technology	Candidate or genome-wide	Number of hits	Reference
Pre-rRNA processing factors	Northern blotting in HeLa cells following treatment with siRNAs	Candidate	286	[11]
40S maturation factors	Imaged-based screening of tetracycline-inducible RPS2-YFP HeLa cells following treatment with siRNAs	Genome-Wide	302	[12]
Nucleolar number	Imaged-based screening of MCF10A cells following treatment with siRNAs and co-staining with anti-fibrillarin antibody	Genome-Wide	252	[13,14]
RNA polymerase I interactors	RNA interactome capture (RIC), RNA immunoprecipitations, quantitative mass spectrometry	Candidate	211	[18]
60S maturation factors	Imaged-based screening of tetracycline-inducible RPL29-GFP HeLa cells following treatment with siRNAs	Genome-Wide	310	[15]

Following their group's candidate screen in 2010, Badertscher et al. performed the first genome-wide siRNA screen for human ribosome biogenesis factors [10,12]. Using an image-based, tetracyline-inducible RPS2-YFP system in HeLa cells to assess defects caused by early ribosome biogenesis defects (i.e. nucleolar accumulation, nucleoplasmic accumulation, etc.), this screen yielded 302 hits involved in 40S maturation [12]. Specifically, as RPS2 is a known small ribosomal subunit protein, defects in SSU maturation factors could be fluorescently detected following gene silencing with siRNAs [12]. Several years later, Dörner et al. conducted a similar screen for 60S maturation factors using known LSU protein RPL29 instead of RPS2. This time, using tetracycline-inducible RPL29-GFP HeLa cells to capture large subunit maturation defects, they identified 310 hits critical for 60S production [15]. Defects in ribosome biogenesis were further characterized using pulse-labeling with ^33^P-orthophosphate followed by audoradiograpgraphy to capture pre-rRNA intermediates, as well as northern blotting and sucrose gradients for polysome profiling [12,15].

Farley-Barnes and McCann et al. took a different image-based, genome-wide siRNA screening approach by using fluorescent microscopy to segment nucleoli to measure changes in nucleolar number. Some of the most significant hits were examined more closely with biochemical assays to assess rRNA transcription (luciferase assays), pre-rRNA processing (northern blotting), and global protein synthesis (puromycin-incorporation measured by Western blotting). The idea for this approach was based upon previous work showing that changes in ribosome biogenesis factors affected nucleolar number in MCF10A cells [26]. In 2018, Farley-Barnes and McCann et al. first published a dataset with hits causing decreases in nucleolar number [13]. The hits causing an increase in nucleolar number were published in a follow up paper in 2021 by Ogawa et al., which focused on the connection between increased nucleolar number, RNAPI transcription, and the cell cycle [14]. For the purposes of this review, the nucleolar number datasets from Farley-Barnes/Ogawa will be combined for a total of 252 hits since they are originally from the same screen [13,14] (screens reviewed in Table 1).

The screens detailed above work in tandem with a better understanding of the nucleolar proteome. The major site of ribosome biogenesis is the nucleolus. Nucleoli form around the tandem repeats ribosomal DNA (rDNA) loci located on the acrocentric chromosomes [13–15,21,22,27,28]. The nucleolus itself is comprised of three compartments, through which the process of ribosome biogenesis moves-the fibrillar center (FC), the dense fibrillar component (DFC), and the granular component (GC) [29]. Understanding the contents of the human nucleolus was a step towards better understanding the process of human ribosome biogenesis. Thus, directed proteomics analyses were undertaken to identify the proteins residing in the nucleolus, alongside more global proteomic analyses [16,17,30–34]. From one of those datasets, the Nucleolar Protein Database (NOPdb) was generated and made publicly available to catalog the nucleolar proteome. (a note: https://www.lamondlab.com/NOPdb3.0/ has been unavailable for over a year and a saved copy has been used for the present analysis). In 2020, Stenström et al. mapped the nucleolar proteome during interphase and mitosis using antibody-based microscopy [35]. These data have been added to the Human Protein Atlas (HPA). For the purposes of this review, the NOPdb and Stenström datasets have been combined for the following comparisons to high-throughput screening datasets in order to capture as many possible known nucleolar proteins.

**Figure 1:**
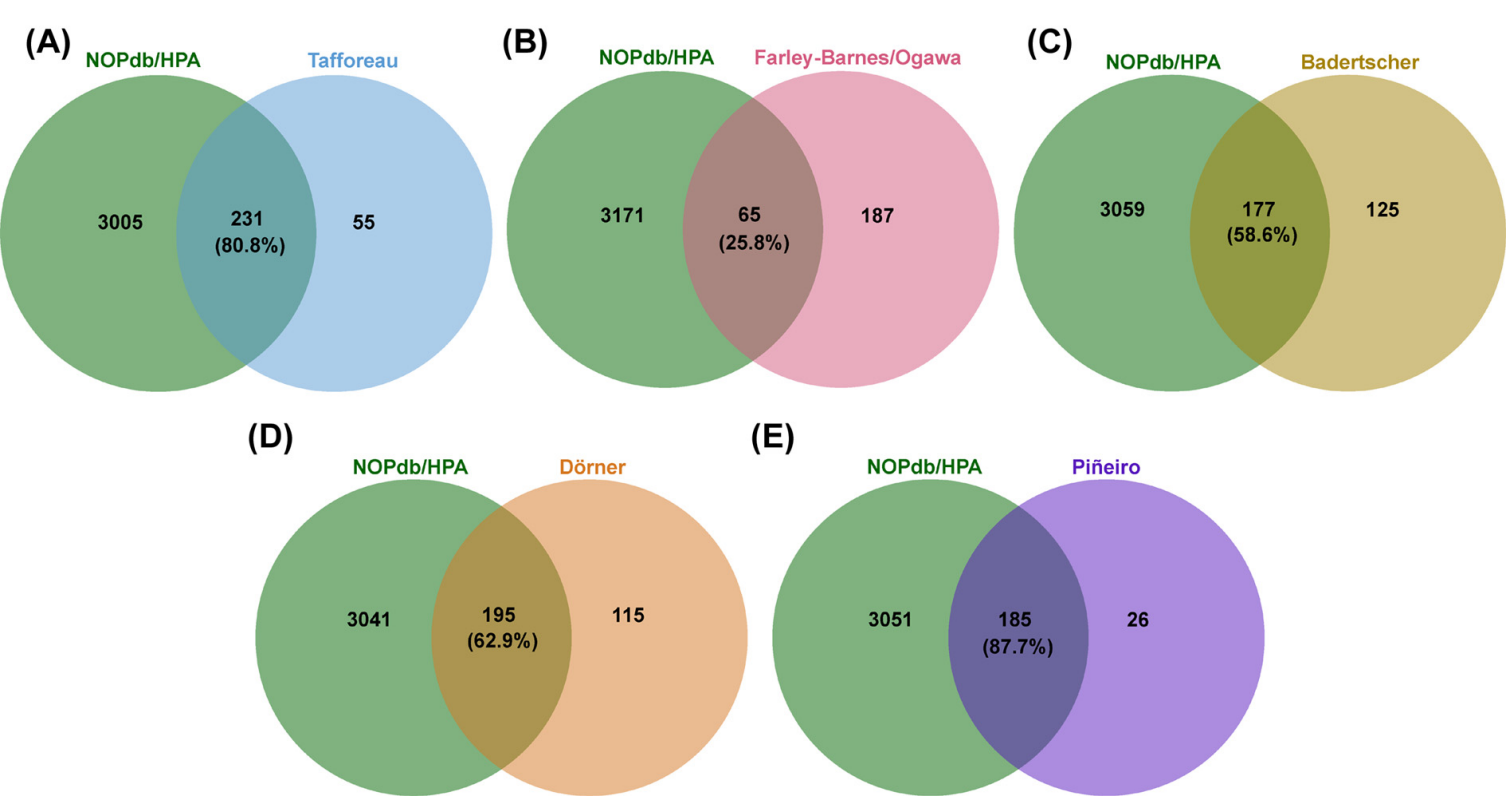
Overlap of candidate genes between screens identifying novel human ribosome biogenesis factors and the Nucleolar Protein Database (NOPdb) and the Stenström et al. dataset (denoted as HPA for the Human Protein Atlas) The contents of the NOPdb from 2009 and the Stenström et al. [35] dataset (denoted as HPA for the Human Protein Atlas) were compared to the contents of (**A**) Tafforeau [11], (**B**) Farley-Barnes/Ogawa [13,14], (**C**) Badertscher [12], (**D**) Dörner [15], and (**E**) Piñeiro [18] using jvenn [36]. The percentage of hits from each dataset found in the NOPdb are annotated under the total number. The datasets for all five screens, and the Stenström et al. dataset, were accessed directly from their respective journal's supplemental material. Given that NOPdb is inaccessible, a 2009 saved copy of the 2008 updated database was used for this analysis [17].

When looking at the datasets from the current screens and a 2009 version of the NOPdb and the Stenström (HPA) dataset, we can see differences in the number of hits present in the nucleolar proteome and each individual screen (Figure 1). The highest percentage of hits present in NOPdb/HPA resides in the Piñeiro screen, followed by the Tafforeau screen, the Dörner screen, the Badertscher screen, and the Farley-Barnes/Ogawa screen with the lowest percentage. The two highest percentages (with 87.7% and 80.8%, respectively), make sense, as the former utilized immunoprecipitations specifically looking for RNA polymerase I interactors (RNA polymerase I resides in the nucleolus), and the latter curated a candidate list based on nucleolar lists of proteins [11,18]. Further, although Farley-Barnes/Ogawa exhibits the lowest percentage of known nucleolar proteins, this actually does also make sense when thinking about the screen readout. A genome-wide screen looking for changes in nucleolar number in comparison to, say, changes in ribosomal RNA processing is bound to pick up more non-nucleolar proteins than not. Changes in nucleolar number could also simply be more affected by non-nucleolar proteins or proteins with major functions outside of ribosome biogenesis than the biological readouts in the other screens, which more specifically capture ribosome biogenesis factors. Excitingly, this screen opens avenues for non-nucleolar proteins and novel pathways involved in ribosome biogenesis. Exploring these non-nucleolar proteins could lead to discoveries about the formation of the nucleolus.

**Figure 2:**
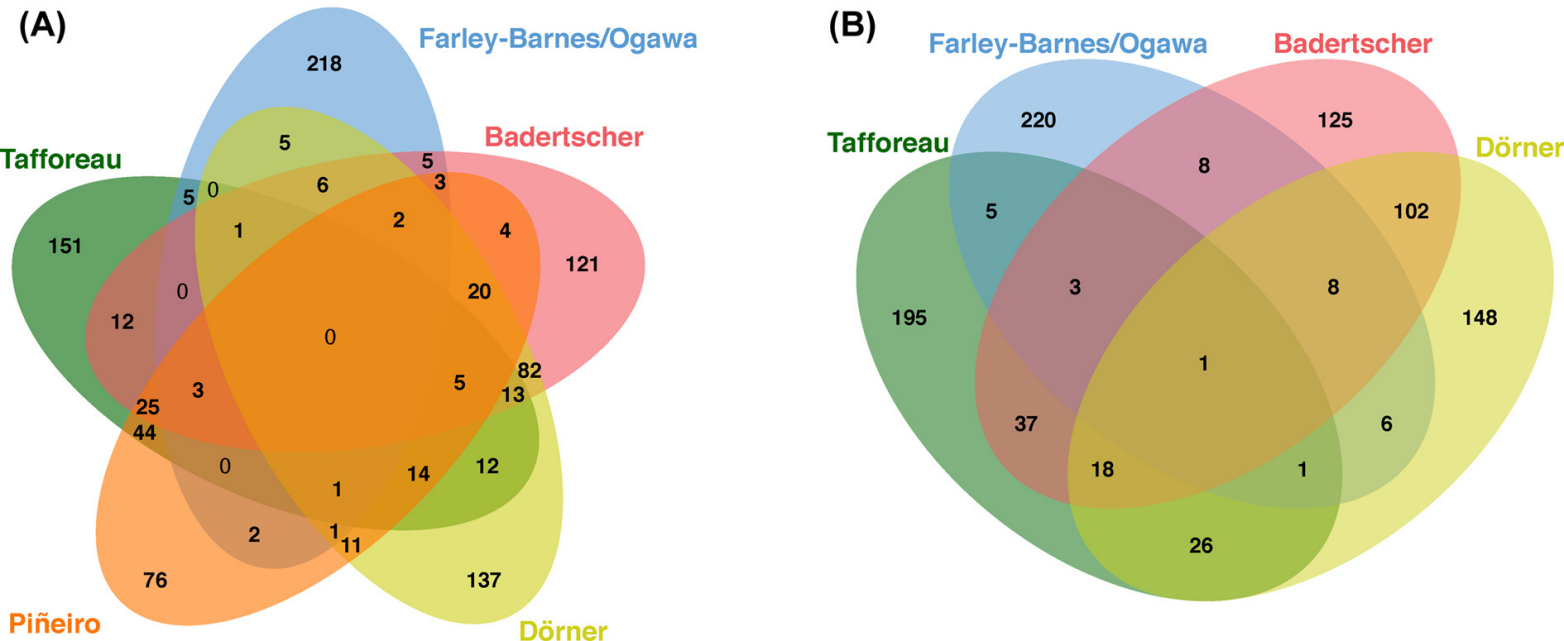
Comparison of screen datasets to each other reveals little to no overlap among them The following datasets were compared using jvenn [36]: (**A**) Tafforeau (green), Farley-Barnes/Ogawa (blue), Badertscher (red), Dörner (yellow), and Piñeiro (orange); (**B**) Tafforeau (green), Farley-Barnes/Ogawa (blue), Badertscher (red), and Dörner (yellow). The datasets for all five screens were accessed directly from their respective journal's supplemental material [11–15,18].

Comparisons between screens allow us to see how the scope and technological approach between studies capture different glimpses of ribosome biogenesis, as well as interesting points where these datasets overlap. Most strikingly, not one hit overlaps in any of the five of the datasets (Figure 2A). However, if the RNAPI interactome study is removed, a single protein overlaps in all of the screens explicitly looking for ribosome biogenesis factors-RPS11 (Figure 2B). However, RPS11 was used as a control in the rRNA processing screen, so it is not a hit although it of course produces a processing defect when depleted. RPS11 is a small subunit ribosomal protein, which means, in part, the 40S maturation factors are readily assessed using these different endpoints. RPS11 was one of the proteins in O'Donohue et el. that fell into the pre-rRNA processing defect category; its knockdown resulted in a failure to process the 5’ETS and ITS1 [8]. Further, a mass spectrometry-based analysis of 26S proteasome non-ATPase regulatory subunit 9 (PSMD9) could also have useful information [37]. RPS11 was among the ribosomal proteins found in the PSMD9 immunoprecipitation complex and thus a part of its interactome [37]. Most strikingly, PSMD9 knockout cell lines showed abnormal nucleolar morphology and induction of nucleolar stress through the stabilization of p53 [37]. Perturbations of RPS11 are at the very least able to be detected by northern blotting, abnormal 40S/60S maturation, and nucleolar number changes, which could indeed make sense if its role has more to do with nucleolar integrity.

The bulk of the substantial overlap occurs between two or three individual screens, with Farley-Barnes/Ogawa being the exception given it did not have substantial overlap with any of the other screens' datasets (<10%). There are a few potential reasons for the differences in hitlists, namely, the different technologies and endpoints being measured, the differences in the siRNA libraries being used, and hit-calling cutoff stringency. The Farley-Barnes/Ogawa dataset also includes more non-nucleolar factors, which affects the comparisons. Further, three of the five screens being evaluated were performed in HeLa cells (Tafforeau, Badertscher, Dörner), while two (Farley-Barnes/Ogawa and Piñeiro) were performed in MCF10A cells, which could also explain some differences between the datasets. NOPdb was also derived from information gleaned from HeLa cells; however, the proteomic content of the human T-cell nucleolus was also mined, and the HPA is based on different cell lines [34,38]. All three nucleolar proteomic datasets were used to assess the number of nucleolar proteins in Farley-Barnes/Ogawa, which still showed a larger subset of non-nucleolar hits in their own analysis [13,14]. Again, the readout of changes in nucleolar number is likely more affected by non-nucleolar proteins than the readouts in the other screens, which might also account for a lack of overlap between the different screens as well.

An interesting throughline arose from these screens-how large and small ribosomal proteins overlapped in the datasets. Farley-Barnes and McCann et al. showed that, except for large ribosomal protein RPLP2, screening for nucleolar number defects only yielded small subunit proteins [13]. RPLP2 is a member of the acidic ribosomal protein P complex that makes up the ribosomal stalk-other members include RPLP0 and RPLP1, with RPLP1 and RPLP2 having two copies in heterodimers that bind to the C-terminus of RPLP0 [39]. This stalk is important for binding translation elongation factors [40]. Depletion of RPLP2 resulting in a decrease in nucleoli could suggest another function perhaps earlier in ribosome biogenesis; however, it was not one of the hits selected for further analysis. The most likely outcome is a protein synthesis defect given its role in translation elongation, but that does not rule out other possible functions. The most similar image-based screening, the iNo scoring, suggested the opposite in that nucleolar morphology was more affected by large subunit proteins [41,42]. Though image-based, the iNo score differs from changes in nucleolar number in that it took the sum of the scores of specific changes in nucleolar morphology as a result of siRNA-mediated gene silencing. Thus, the differences in the predominance of large compared with small subunit proteins can likely be attributed to the readout from each screen: nucleolar number versus nucleolar morphology, as well as the basic differences between screens described above. Furthermore, Farley-Barnes and McCann et al. used a known SSU factor, UTP4, as the positive control in their experiments, which could bias the screen towards an SSU saturation [13]. The Kutay group similarly noted that strong defects in large subunit processing affected small subunit processing, but the reciprocal effect was not seen [10,12]. This effect on SSU factors from LSU factors, but not the inverse, has been previously observed [43–47]. A follow-up report on a hit (RSL24D1) from the Farley-Barnes/Ogawa dataset, which also appeared in the Tafforeau and Piñeiro datasets, further showcased the connection between LSU processing and earlier steps in ribosome biogenesis, namely rRNA transcription [48]. Specifically, RSL24D1 is important for pre-rRNA and mature 28S rRNA processing, as well as the regulation of steady-state RPA194 levels [48]. Finally, the structure of the nucleolus at least in part appears to be maintained by LSU factors and their interaction with nucleophosmin 1 (NPM1) dictating the volume of the nucleolus [49]. Overall, there seems to be a strong connection between the large ribosomal subunit factors and nucleolar form.

Another point of overlap between datasets from screens and research on specific ribosome regulators is the protein Che-1/AATF (apoptosis antagonizing transcription factor), an important DNA damage response target [50]. Piñeiro et al. found AATF as a part of the RNAPI interactome that binds to the 45S pre-rRNA [18]. AATF was also found to localize to the nucleolus on the available proteomes, including the NOPdb, and it was a hit in both the Tafforeau and Badertscher screens, and shown to be important for pre-rRNA processing and 40S assembly, respectively [11,12]. At the same time, other groups also using RNA interactome capture (RIC, like Piñeiro et al.) identified AATF as an RNA-binding protein, with Kaiser et al. confirming 45S pre-rRNA as the most enriched transcript, among other ribosomal RNAs [50–56]. In fact, 15% of their 160 interactors were ribosomal proteins, with AATF implicated as a major factor in SSU processing [56]. These data were consistent with previous reports of a depletion of AATF resulting in a defect in early pre-rRNA precursors [57].

The amount of data we have from former and ongoing screens has greatly increased our understanding of ribosome biogenesis. Analyzing the datasets and discovering the overlapping and non-overlapping areas extends our understanding and shows us where there is perhaps room for expansion in the high-throughput screening market. We still have much left to explore with regards to identifying the full spectrum of human ribosome biogenesis factors and how they might interact with one another.

## Areas to enhance our understanding of human ribosome biogenesis factors

Even though the field has a diverse set of screens to date, the endpoints do clearly affect the overall saturation of possible hits, as do the libraries used for the initial gene silencing. Two endpoints that have not been used in a primary screen are the transcription of the ribosomal RNA and the ultimate output of ribosome biogenesis, protein synthesis. Furthermore, the landscape of regulation far surpasses the classical protein-coding genomic regions [58–60]. Great work has been done to probe these specific steps of ribosome biogenesis and derive important understandings that could be re-applied differently to extend previously held findings. The advent of newer technologies, or older technologies applied in new ways, has also allowed for exciting advance in better characterizing human ribosome biogenesis.

Though the RNAPI interactome generated valuable information, further understanding RNAPI activity and its other regulators is also necessary [61–64]. This information also requires a mapping of the mechanism of RNAPI interactors to fully understand their potential roles in ribosomal DNA transcription. Piñeiro et al. confirmed the specific binding interaction of two proteins-AATF and NGDN-with the pre-rRNA in a targeted immunoprecipitation experiment. In order to expand our knowledge of transcriptional regulators, we could use rRNA transcription specifically as an endpoint in a high-throughput screen. The pre-rRNA transcript has previously been measured by qRT-PCR as a secondary screen in a high-throughput platform [65]. The primary screen was an imaged-based, Halo-RPS9 measurement in A375 cells, similar to the Badertscher and Dörner platforms, with the top hits being subjected to qRT-PCR to identify hits that affect pre-rRNA levels. This particular screen was looking for compounds that impacted ribosome biogenesis, but could easily be applied to a genome-wide screen for protein or other cellular regulators of ribosome biogenesis. Specifically, following genome-wide depletion of proteins, RNAPI TaqMan qRT-PCR could be used to determine proteins involved in RNAPI transcription of the pre-rRNA transcript. Another measurement of nucleolar RNA biogenesis comes is 5-ethynyl uridine incorporation, which has been used previously to measure nucleolar RNA production and was further modified for a more high-throughput method [66,67]. Thus, the technology is there to specifically look for regulators of rRNA and it would be helpful to have these datasets to compare against those of Piñeiro.

Much like ribosomal RNA transcription, global protein synthesis has not been used as an endpoint in a primary screen for protein regulators of ribosome biogenesis. Since impaired ribosome biogenesis diminishes global protein synthesis in a cell, nascent translation could potentially be harnessed as an observable for ribosome biogenesis screens. A recent screening campaign used o-propargyl-puromycin (OPP) and click chemistry to visual proteins following chemical perturbation [68]. Puromycin is an aminoacyl tRNA analog and OPP is a puromycin analog that can be conjugated to fluorophores and quantitated [69,70]. This screen was actually only the third of its kind, marking fertile ground for future works in the realm of ribosome biogenesis [71,72]. Again this campaign was used for drug screening, but Western blot analysis of puromycin-incorporation has successfully been used to determine a role for a protein in ribosome biogenesis, which also makes this technology attractive for high-throughput screening [13,14,73]. Related to using qRT-PCR as an endpoint, using OPP and click chemistry after gene silencing, followed by fluorescent quantitation to measure protein synthesis, could identify important proteins involved in protein synthesis. Muller et al. similarly used succinimidyl ester conjugated to a fluorophore and click chemistry to assess total protein content in high-throughput, in conjunction with nucleolar segmentation [66]. Protein synthesis as an endpoint for ribosome biogenesis factor identification, however, is also problematic in that other processes besides ribosome biogenesis can affect protein synthesis [74]. This type of screen would necessitate a secondary screen probing more specific ribosome biogenesis steps.

Finally, the bias towards proteins in present studies does mean that other aspects of ribosome biology have been unexplored. This includes miRNAs and lncRNAs, which have become increasingly recognized as layers of regulation in ribosome biogenesis [58,75,76]. Our understanding of lncRNAs in particular is becoming clearer but still very much lags behind proteins. Even supposed noncoding elements could perhaps encode micropeptides [77]. Protein-coding sequences themselves can also contain small unannotated open reading frames (smORFs) [59,60]. Cao et al. used a biorthogonal protein labeling of cysteine sites with proteomics to identify alt-RBM10 (MINAS-60), a late 60S assembly inhibitor [78]. An alternative protein (alt-protein) can arise from a frame-shifted ORF overlapping a coding sequence, with the coding sequence for MINAS-60 being for RBM10, typically involved in splicing and upregulated in certain cancers [79,80]. Another alt-protein, alt-Laminin subunit alpha-3 (alt-LAMA3), was discovered using TurboID, a proximity labeling technique using an engineered biotin ligase, that allowed for subcellular compartment assessment [81]. Alt-LAMA3 was found to interact with the PeBoW complex, which is associated with pre-rRNA processing and transcription, and to be required for pre-rRNA transcription and protein synthesis [48,81–84]. There are many smORFs and alt-proteins waiting to be discovered, some of which may be involved in ribosome biogenesis [59].

## Using screens for clinical applications

Aside from discovery of novel regulators of ribosome biogenesis, screens can also directly search for compounds that alter nucleolar function. Ribosome synthesis and the nucleolus itself have become attractive targets for potential therapies against ribosomopathies and cancer [61,85,86]. A number of screens have elucidated roles of well-known chemotherapeutic agents in ribosome biogenesis, as well as aided in the discovery of new drugs with the potential to treat human disease [87–92].

p53 is a well-known tumor suppressor implicated in a host of cancers and is well-connected to the nucleolus in terms of the nucleolar stress response [93–95]. Peltonen et al. undertook a high-content image-based screening campaign to identify p53 activating compounds [89]. Using a p53 reporter and imaging followed by secondary assays for mechanistic studies, the lead compound, BMH-21, was found to inhibit rDNA transcription by intercalating with GC rich rDNA, leading to the dissociation and degradation of RPA194 [89,90]. RPA194 is the catalytic subunit of RNAPI. Interestingly, an RNAi screen using immunofluorescence of RPA194 levels led to the discovery of Skp-Cullen-F-box (SCF)^FBXL14^ (FBXL14) as the E3 ubiquitin ligase that targets RPA194 for degradation [96]. The mechanism of protein degradation of RPA194 by BMH-21 also opens up avenues of using technology such as protein-targeting chimeric molecules, or PROTAC, for RNAPI [97]. The potential use of PROTACs comes with certain safety considerations such as off-target protein degradation, prolonged protein degradation, and increasing the concentration of ubiquitinated proteins in the cell, among others [98]. Another RNAi screening campaign for p53 regulators revealed p53 accumulation after depletion of DDX56, WDR75, and BYSL [99]. More specifically, the screen depleted ribosomal binding factors and used an imaged-based microscopy approach to detect fold-changes in p53 levels [99]. This screen confirmed the connection between 40S ribosomal proteins and 5S RNP-dependent p53 activation but not 60S ribosomal subunit assembly [95,99–104]. This screen uncovered a role for WDR75 in maintaining RPA194 levels to aid in ribosomal RNA transcription [99].

Transcription of the ribosomal RNA itself has also been an attractive target for two screens, one of which was discussed above [65]. Drygin et al. compared transcripts from RNA polymerase I to RNA polymerase II following treatment with compounds to determine which small molecules had an effect on RNAPI activity, leading to the discovery of CX-5461 [105]. Further studies showed that CX-5461 acts as a topoisomerase II poison as its mechanism for RNAPI inhibition [106]. The screens to identify compounds that inhibit ribosome biogenesis must be used in conjunction with detailed approaches to fully understand the mechanisms of action. Excitingly, CX-5461 has been used in Phase I clinical trials for BRCA1/2 breast and ovarian patients in Canada [107]. CX-5461 was well-tolerated at the recommended phase II dose (475 mg/m^2^), with anti-tumor activity mainly shown in HR-defective tumors, and a resistance to CX-5461 in conjunction with reversion to wild-type *PALB2* and *BRCA2* (evidence of synthetic lethality of these types of compounds) [107,108]. Screens to identify attractive drug targets combined with screens to identify novel drugs to treat diseases have made large strides in the past two decades, a trend that will hopefully only continue.

## What is the next frontier? Conclusions and perspectives

Many high-throughput experiments have been conducted to understand human ribosome biogenesis in the screens detailed throughout this review, but where do we go from here? An interesting source of connections between the found regulators of ribosome biogenesis and the formation or integrity of the nucleolus have emerged. Furthermore, advances in long-read sequencing have finally allowed for large-scale assemblies of the human genome and a fuller picture of the ribosomal DNA [109,110]. There are so many places to go from here, and groups are already exploring pairing technological advances with discovery-based methods to unlock connections in ribosome biogenesis. We can conduct high-throughput screens to identify non-protein regulators (such as lncRNAs and miRNAs) and non-canonical protein-coding molecules (such as smORFs). Perhaps one of the most interesting new avenues is the use of real-time or live-cell imaging to observe all or parts of ribosome biogenesis in intact cells [111–113]. Matsumori et al. incorporated both live-cell imaging and screening to detect ribosomal proteins that led to malformed nucleoli, and their data deepened the connection between LSU proteins and nucleolar integrity [112]. In the next twenty years, we foresee that additional connections between datasets and technology will continue to boost discovery of new human ribosome biogenesis factors, in addition to already known players into more detailed models describing nucleolar form and function.

## Open Access

Open access for this article was enabled by the participation of Yale University in an all-inclusive *Read & Publish* agreement with Portland Press and the Biochemical Society under a transformative agreement with Individual.

## Abbreviations

alt-LAMA3: alt-Laminin subunit alpha-3
NPM1: nucleophosmin 1
OPP: o-propargyl-puromycin
pre-rRNA: precursor ribosomal RNA
smORF: small open reading frame
